# The Role of the Iron Transporter *ABCB7* in Refractory Anemia with Ring Sideroblasts

**DOI:** 10.1371/journal.pone.0001970

**Published:** 2008-04-09

**Authors:** Jacqueline Boultwood, Andrea Pellagatti, Maryam Nikpour, Beena Pushkaran, Carrie Fidler, Helen Cattan, Tim J. Littlewood, Luca Malcovati, Matteo G. Della Porta, Martin Jädersten, Sally Killick, Aristoteles Giagounidis, David Bowen, Eva Hellström-Lindberg, Mario Cazzola, James S. Wainscoat

**Affiliations:** 1 LRF Molecular Haematology Unit, Nuffield Department of Clinical Laboratory Sciences (NDCLS), John Radcliffe Hospital, Oxford, United Kingdom; 2 Division of Hematology, Department of Medicine, Karolinska Institutet, Stockholm, Sweden; 3 Division of Hematology, University of Pavia Medical School, IRCCS Policlinico S. Matteo, Pavia, Italy; 4 Department of Haematology, Royal Bournemouth Hospital, Bournemouth, United Kingdom; 5 Medizinische Klinik II, St. Johannes Hospital, Duisburg, Germany; 6 Haematology Department, Leeds Teaching Hospitals, Leeds, United Kingdom; Ordway Research Institute, United States of America

## Abstract

Refractory Anemia with Ring Sideroblasts (RARS) is an acquired myelodysplastic syndrome (MDS) characterized by an excess iron accumulation in the mitochondria of erythroblasts. The pathogenesis of RARS and the cause of this unusual pattern of iron deposition remain unknown. We considered that the inherited X-linked sideroblastic anemia with ataxia (XLSA/A) might be informative for the acquired disorder, RARS. XLSA/A is caused by partial inactivating mutations of the *ABCB7* ATP-binding cassette transporter gene, which functions to enable transport of iron from the mitochondria to the cytoplasm. Furthermore, *ABCB7* gene silencing in HeLa cells causes an accumulation of iron in the mitochondria. We have studied the role of *ABCB7* in RARS by DNA sequencing, methylation studies, and gene expression studies in primary CD34^+^ cells and in cultured erythroblasts. The DNA sequence of the *ABCB7* gene is normal in patients with RARS. We have investigated *ABCB7* gene expression levels in the CD34^+^ cells of 122 MDS cases, comprising 35 patients with refractory anemia (RA), 33 patients with RARS and 54 patients with RA with excess blasts (RAEB), and in the CD34^+^ cells of 16 healthy controls. We found that the expression levels of *ABCB7* are significantly lower in the RARS group. RARS is thus characterized by lower levels of *ABCB7* gene expression in comparison to other MDS subtypes. Moreover, we find a strong relationship between increasing percentage of bone marrow ring sideroblasts and decreasing *ABCB7* gene expression levels. Erythroblast cell cultures confirm the low levels of *ABCB7* gene expression levels in RARS. These data provide an important link between inherited and acquired forms of sideroblastic anemia and indicate that *ABCB7* is a strong candidate gene for RARS.

## Introduction

The myelodysplastic syndromes (MDS) are a heterogeneous group of hematopoietic malignancies, characterized by blood cytopenias, ineffective hematopoiesis and a hypercellular bone marrow [1]. Refractory Anemia with Ring Sideroblasts (RARS) is a subtype of MDS in which excess iron accumulates in the form of aberrant mitochondrial ferritin in the mitochondria of the erythroid precursors [2], [3]. RARS is a myeloid malignancy and transforms to acute leukemia in approximately 10–20% of cases [4]. The molecular genetic basis of RARS remains unknown.

The hereditary sideroblastic anemias are a heterogeneous group of disorders characterized by the presence of ring sideroblasts in the bone marrow, microcytic hypochromic anemia and typically show X-linked inheritance [5]–[7]. Many of the reported cases of inherited X-linked sideroblastic anemia have been found to be caused by mutations in the erythroid-specific delta-aminolevulinate synthase gene (*ALAS2*) [8]. A rare inherited X-linked sideroblastic anemia with ataxia (XLSA/A) is caused by mutations of the mitochondrial ATP-binding cassette transporter *ABCB7* [9], [10]. The *ABCB7* gene is the functional orthologue of the yeast Atm1p gene [11], which is required for mitochondrial iron homeostasis [12] and has been implicated in the transport of a component required for the maturation of iron-sulfur (Fe-S) cluster proteins out of mitochondria [13]. It has been demonstrated that XLSA/A is due to partial loss of function mutations in *ABCB7* that directly or indirectly inhibit heme biosynthesis [13]. The anemia in XLSA/A is caused by the accumulation of iron in a form that is not readily usable for heme synthesis [14]. Moreover, ABCB7 has been demonstrated to be essential for hematopoiesis in experiments using conditional gene targeting in mice [13]. In an important experiment, *ABCB7* has been silenced in HeLa cells by sequential transfection experiments with siRNAs [14]. The phenotype of the *ABCB7*-deficient cells was characterized by a reduction in proliferation rate that was not rescued by iron supplementation, by signs of iron deficiency, and by a large increase of iron accumulation in the mitochondria that was poorly available to mitochondrial ferritin.

We have investigated the expression levels of the *ABCB7* gene using data from our large microarray dataset on CD34^+^ cells from MDS patients and healthy controls in order to determine whether the *ABCB7* gene is implicated in the common acquired sideroblastic anemia, RARS.

## Materials and Methods

### Sample collection and cell separation

122 patients with MDS and 16 healthy controls were included in the study. Classification of MDS patients was according to the French-American-British (FAB) criteria [15] and the patients were selected solely on the basis of having MDS. The MDS patient samples were obtained from several sources: Oxford, Bournemouth and Dundee (United Kingdom), Stockholm (Sweden), Duisburg (Germany) and Pavia (Italy). At the time of investigation, 35 patients had Refractory Anemia (RA), 33 RARS and 54 Refractory Anemia with Excess Blasts (RAEB). The study was approved by the ethical committees of the John Radcliffe Hospital (Oxford), the Karolinska Institutet (Stockholm), the IRCCS Policlinico S. Matteo (Pavia), the Royal Bournemouth Hospital (Bournemouth), the St Johannes Hospital (Duisburg) and the Ninewells Hospital (Dundee); written informed consent was obtained from study participants. CD34^+^ cells were isolated from the bone marrow samples of MDS patients and healthy controls using MACS magnetic cell separation columns (Miltenyi Biotec, Bergisch Gladbach, Germany) according to the manufacturer's recommendations.

### Erythroblast cell cultures

CD34^+^ cells isolated from the bone marrow of 8 patients with RARS, 9 patients with RA and 8 healthy controls were cultured according to a method developed to study the generation of erythroblasts [16], [17]. Gene expression profiling experiments were performed on cultured cells at day 7.

### Microarray experiments and data analysis

Gene expression profiling experiments and data analysis were performed as previously described [18]. Affymetrix GeneChip Human Genome U133 Plus 2.0 arrays (47,000 transcripts) were used. Gene correlations were calculated using Pearson correlation.

### Real-time quantitative PCR

Real-time quantitative PCR for the *ABCB7* gene was performed using cDNA made from the unamplified RNA from CD34^+^ cells of 7 patients with RARS, 8 patients with RA, 6 patients with RAEB and 7 healthy controls. Beta-2-microglobulin was used to normalize for differences in input cDNA. Reactions were performed as previously described [16] and expression ratios were calculated using the ΔΔC_T_ method [19].

### Statistical analysis

Statistical analysis and box plots were created using StatView software. Kruskal-Wallis test was used for comparison between three or more groups and Mann-Whitney U test for comparisons between any two groups. A P-value <0.01 was considered significant.

### DNA sequencing

Direct sequencing of *ABCB7* was performed on DNA from 13 RARS patients using Applied Biosystems Big dye terminator kit v1.1.

### Promoter methylation studies

We looked at promoter methylation of *ABCB7* in DNA samples from 6 RARS patients (2 bone marrow, 2 neutrophil and 2 CD34^+^ samples) and from 5 healthy controls (1 bone marrow, 1 neutrophil, 1 mononuclear cell and 2 CD34^+^ samples). The *ABCB7* promoter was predicted using PromoterScan and CpGPlot software. For investigation of methylation status we used bisulphite sequencing. Bisulphite modification of the genomic DNA was carried out using the EpiTect Bisulphite kit (Qiagen). Primers to amplify the modified DNA for bisulphite sequencing were designed using Primer3 software and were as follows: 5′-GTTTGGGGTTTTGGGAAAAT-3′ (*ABCB7* forward primer) and 5′-TTCCATATCAATAACATCTCACCTAA-3′ (*ABCB7* reverse primer). The PCR products were purified with the Wizard PCR Clean-Up System and Vacuum Manifold (Promega, Madison, WI) according to the manufacturer's instructions. PCR products were cloned using the pGEM-T Easy cloning system (Clontech) and sequenced with Applied Biosystems Big dye terminator kit v1.1 and the *ABCB7* forward primer.

## Results

### ABCB7 expression levels in MDS subtypes

**Figure 1:**
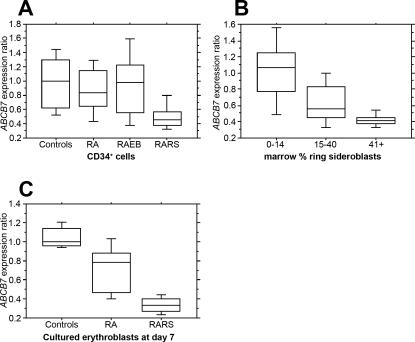
*ABCB7* expression levels. (A) *ABCB7* expression levels in the CD34^+^ cells from healthy controls and MDS patients with RA, RAEB and RARS. (B) *ABCB7* expression levels and percentage of ring sideroblasts in the bone marrow. (C) *ABCB7* expression levels in cultured erythroblasts from healthy controls and MDS patients with RA and RARS. The boxes represent the 25^th^, 50^th^ and 75^th^ percentiles, the bars correspond to the 10^th^ and 90^th^ percentiles.

We have determined the *ABCB7* gene expression levels from our microarray dataset on CD34^+^ cells in 122 MDS patients and 16 healthy controls. We have compared the gene expression levels of *ABCB7* between 35 patients with refractory anemia (RA), 33 patients with RARS, 54 patients with RA and excess blasts (RAEB), and 16 healthy controls (Table 1; Figure 1A). The expression levels of *ABCB7* are lowest in the RARS group compared to RA (p<0.0001), RAEB (p<0.0001) and healthy controls (p<0.0001). No significant difference was observed between the RA, RAEB and healthy control groups.

**Table 1:** Descriptive statistics of *ABCB7* expression levels in CD34^+^ cells according to FAB group, in CD34^+^ cells according to percentage of bone marrow ring sideroblasts, and in cultured erythroblasts at day 7.

CD34^+^ CELLS - FAB	MEAN	STD DEV	Kruskal-Wallis p<0.0001			
Controls (n = 16)	0.98	0.36		Controls	RA	RAEB
RA (n = 35)	0.88	0.34	RA	0.3610	—	—
RAEB (n = 54)	0.94	0.44	RAEB	0.5476	0.7211	—
RARS (n = 33)	0.49	0.19	RARS	<0.0001	<0.0001	<0.0001
**% SIDEROBLASTS**	**MEAN**	**STD DEV**	**Kruskal-Wallis p<0.0001**			
0–14 (n = 57)	1.03	0.39		0–14	15–40	
15–40 (n = 26)	0.62	0.26	15–40	<0.0001	—	
41+ (n = 18)	0.42	0.07	41+	<0.0001	0.0022	
**DAY 7 ERYTHROBLASTS**	**MEAN**	**STD DEV**	**Kruskal-Wallis p = 0.0001**			
Controls (n = 8)	1.05	0.11		Controls	RA	
RA (n = 9)	0.73	0.24	RA	0.0071	—	
RARS (n = 8)	0.34	0.08	RARS	0.0008	0.0021	

### ABCB7 expression levels and percentage of bone marrow ring sideroblasts

The 101 of 122 MDS cases, where data was available on the exact percentage of bone marrow ring sideroblasts, have been divided in three groups by percentage of bone marrow ring sideroblasts (0–14%, 15–40% and >41%) (Table 1; Figure 1B). It can be seen that the median expression level of *ABCB7* falls progressively across the three groups. As shown in Table 1, the differences in *ABCB7* expression between the three groups are all statistically significant.

### ABCB7 expression levels in erythroblast cultures

In addition, we have studied the *ABCB7* gene expression levels in erythroblast cultures derived from the CD34^+^ cells of 8 patients with RARS, 9 patients with RA and 8 healthy controls. Cells were cultured according to a method developed to study the generation of erythroblasts [16], [17] and gene expression profiling experiments were performed on cultured cells at day 7. We confirmed the low levels of *ABCB7* gene expression levels in RARS erythroblasts in comparison to those of RA and healthy controls (Table 1; Figure 1C).

### Confirmation of ABCB7 expression data

**Figure 2:**
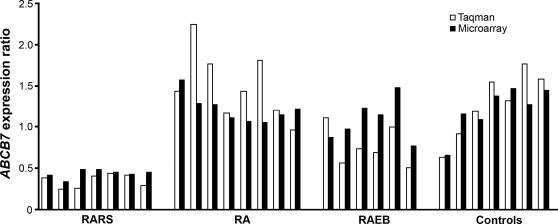
Confirmation of the microarray data for the *ABCB7* gene using real-time quantitative PCR.

The microarray-generated expression data of the *ABCB7* gene were validated and confirmed in a subset of 21 MDS patients (7 patients with RARS, 8 patients with RA, 6 patients with RAEB) and 7 healthy controls using real-time quantitative PCR (Figure 2). A high concordance was observed between the expression levels obtained with Affymetrix chips and with real-time quantitative PCR, indicating a good level of agreement between the two assays.

### ABCB7 gene sequencing and promoter methylation

Direct sequencing of *ABCB7*, including the promoter region, was performed on DNA from 13 RARS patients and did not show any mutations in any of the patients. We have investigated the methylation status of the *ABCB7* promoter region in 6 patients with RARS and 5 healthy controls, and the results showed >80% methylation in all samples, with no significant difference between RARS patients and healthy controls.

### ABCB7 gene correlation

No genomic abnormality in the *ABCB7* gene was identified in our cases. Therefore, we examined our microarray dataset for genes with expression levels correlated either strongly positively (≥0.8) or inversely (≤−0.8) with *ABCB7* gene expression levels. The expression of two genes showed a positive correlation with *ABCB7* expression: *ELP2* (0.82) and *RTF1* (0.80). No genes were found whose expression correlated inversely with *ABCB7*.

## Discussion

We have examined the role of *ABCB7*, the gene involved in the hereditary XLSA/A, in the acquired RARS syndrome. The hypothesis under consideration is that XLSA/A and acquired RARS may have a shared molecular basis. Interestingly, there is an excess of erythrocyte protoporphyrin in XLSA/A (in contrast with other types of genetic sideroblastic anemia) as also found in RARS [20]. We have determined the *ABCB7* gene expression levels from our microarray dataset on CD34^+^ cells in 122 MDS patients and 16 healthy controls. The expression levels of *ABCB7* are significantly lower in the RARS group than in healthy controls, RA or RAEB. In contrast there is no significant difference in *ABCB7* expression between the latter three groups.

The definition of RARS includes more than 15% ring sideroblasts in the bone marrow. However, we find not only lower levels of *ABCB7* in RARS than other MDS but also a progressive reduction of *ABCB7* expression as the percentage of ring sideroblasts increases. In addition, we have studied the *ABCB7* gene expression levels in erythroblast cultures and confirmed the low levels of *ABCB7* gene expression levels in RARS erythroblasts in comparison to those of RA and healthy controls.

The gene expression levels of *ABCB7* were validated and confirmed in a subset of MDS patients and healthy controls using real-time quantitative PCR. Direct sequencing of *ABCB7*, including the promoter region, was performed on DNA from 13 RARS patients and did not show any mutations in any of the patients. Similarly, Steensma *et al*, in a recent study, found no coding mutations of *ABCB7* in patients with RARS [20].

We have investigated the methylation status of the *ABCB7* promoter region and the results showed >80% methylation in all samples, with no significant difference between RARS patients and healthy controls. Gene promoters can be divided into three groups according to their CpG ratio, GC content and length of CpG-rich region: high-CpG, intermediate-CpG and low-CpG promoters [21]. Based on this classification, the *ABCB7* promoter region is defined as an intermediate-CpG promoter. Intermediate-CpG promoters show a high frequency of DNA methylation [21]. Our data fit with the definition of the *ABCB7* promoter region as an intermediate CpG promoter, but suggest that promoter methylation is unlikely to be the mechanism underlying the reduction in expression levels of *ABCB7* observed in RARS.

No genomic abnormality in the *ABCB7* gene was identified in our cases, suggesting that there could be a trans-acting factor responsible for the low levels of *ABCB7* in RARS (analogous to the down regulation of the alpha-globin genes in the acquired HbH associated with MDS caused by mutations in the *ATRX* gene) [22]. Therefore, we examined our microarray dataset for genes with expression levels correlated either strongly positively (≥0.8) or inversely (≤−0.8) with *ABCB7* gene expression levels. While no other gene had a strong inverse correlation with *ABCB7*, interestingly two genes showed a positive correlation: *ELP2* (0.82) and *RTF1* (0.80), both of which are associated with RNA Polymerase II [23], [24]. Deregulation of these factors may cause defective gene transcription through chromatin [25].

The existence of an inherited disorder XLSA/A caused by inactivating mutations in *ABCB7* is, of course, very informative of the probable consequences of reduced expression of the same gene in acquired disorders. Many of the key properties of ABCB7 have already been established by gene silencing studies and by conditional gene targeting in mice [13], [14]. All of these experiments are consistent with the importance of ABCB7 in hematopoiesis and with the observed phenotype in XLSA/A. Therefore, it would be predicted that erythroblasts with low *ABCB7* levels would have abnormal mitochondrial iron homeostasis. Our finding of a strong association between RARS and low gene expression levels of *ABCB7* also raises the possibility of therapeutic approaches for RARS using drugs which induce the expression of ABCB7, for example, carbamazepine has been shown to induce ABCB7 in the liver of epileptic patients treated with the drug [26]. We suggest that down-regulation of the iron transporter *ABCB7* plays an important role in the molecular pathogenesis of RARS, making an intriguing link between the inherited and acquired forms of sideroblastic anemia.
